# Response: Commentary: The ME-BYO index: a development and validation project of a novel comprehensive health index

**DOI:** 10.3389/fpubh.2025.1654394

**Published:** 2025-11-06

**Authors:** Hiroto Narimatsu, Sho Nakamura, Ryo Watanabe, Yoshinobu Saito, Kaname Watanabe, Ung-il Chung

**Affiliations:** 1 Graduate School of Health Innovation, Kanagawa University of Human Services, Kawasaki, Japan; 2 Cancer Prevention and Control Division, Kanagawa Cancer Center Research Institute, Yokohama, Japan; 3 Center for Innovation Policy, Kanagawa University of Human Services, Kawasaki, Japan; 4 Department of Genetic Medicine, Kanagawa Cancer Center, Yokohama, Japan; 5 Faculty of Sport Management, Nippon Sport Science University, Yokohama, Japan; 6 Department of Bioengineering, Graduate School of Engineering, The University of Tokyo, Tokyo, Japan

**Keywords:** preventive medicine and public health, healthy aging, index, smartphone, quality of life

The ME-BYO Index is a comprehensive health assessment tool measuring the dynamic transition between health and disease states. Core domains encompass metabolic function, locomotor function, cognitive function, and mental resilience, all of which contribute to personal health management. Regarding the alignment with the WHO framework, the ME-BYO index's four domains directly correspond to the WHO's Intrinsic Capacity components: locomotor function aligns with physical mobility, metabolic function with vitality, cognitive function remains directly matched, and mental resilience encompasses psychosocial aspects, while sensory capacity represents an area for future expansion.

We extend our sincere gratitude to Ikewaki et al. (1) for their insightful commentary on our article concerning the development of the ME-BYO index (2). We appreciate their valuable suggestions, particularly regarding the potential role of the neutrophil-to-lymphocyte ratio (NLR) as an immune marker and the application of beta-glucans as biological response modifiers (BRMGs). These perspectives are significant in developing a more specific or customized ME-BYO index. As highlighted in their commentary, the NLR reflects immune balance and systemic inflammation, and its inclusion could contribute a valuable biological dimension to the ME-BYO index, indicating promising avenues for preventive interventions aligned with the ME-BYO concept's focus on early-stage health transitions. However, its limitations must be noted; thresholds vary according to the clinical setting, and significant confounding factors, such as age, comorbidities, and medications, may affect the testing results.

We propose implementing a three-tier architecture for ME-BYO indicators. Tier 1 (Core Self-Administered Indicators) are the foundational components of the current ME-BYO framework—cognitive assessment (Mini-Cog), locomotor function evaluation (Locomo-5 questionnaire and walking speed), mental resilience measurement (MIMOSYS voice analysis), and basic health data—all of which are deliverable through smartphone applications to ensure broad population accessibility. Tier 2 (Laboratory Add-On Indicators) incorporates clinical laboratory parameters such as NLR within healthcare settings. Tier 3 (Research-Grade Indicators) encompasses emerging biomarkers and experimental interventions, such as beta-glucans, that require rigorous validation before they can be implemented clinically. This tiered approach maintains ME-BYO's core accessibility while enabling enhanced biological depth in appropriate contexts, supporting evolution from population health screening to precision medicine applications.

It is worth noting that the commentary's discussion of β-glucans necessitates careful consideration of sources. Immune-modulating 1,3/1,6 β-glucans from yeast and fungal sources exhibit fundamentally different mechanisms compared to cereal-derived 1,3/1,4 β-glucans, which are primarily recognized for their lipid-lowering effects. While the reported impact on NLR modulation and immune function represents promising preliminary findings, significant heterogeneity exists across fungal strains, extraction methods, dosing protocols, and clinical outcomes, making broad generalizations scientifically inappropriate without strain-specific validation.

In addition to biological indicators, we wish to emphasize our research team's ongoing efforts to incorporate efficiency metrics based on Data Envelopment Analysis (DEA) into population-level health assessments. We utilized DEA to evaluate the potential risk of non-communicable disease onset, thereby providing an objective methodology to assess specific indicators in health interventions (3, 4). The DEA efficiency is calculated using the Charnes-Cooper-Rhodes model with specific input variables (inverse of salt/energy intake, physical activity) and output variables (inverse of blood pressure/lipid values). Scores range from 0.0 to 1.0, with higher values indicating better efficiency (lower future disease risk). More recently, we conducted a randomized controlled trial employing an efficiency score derived from the DEA to identify high-risk individuals for targeted primary preventive interventions, which was published in a non-peer-reviewed paper (5). Future development plans include the application of DEA methodology to enable individualized disease risk assessment with enhanced sensitivity and specificity. DEA would allow the system to identify personalized efficiency frontiers by comparing each individual's health profile against optimal performance benchmarks derived from similar demographic and clinical characteristics, thereby providing more nuanced risk stratification than current composite scoring. This DEA-enhanced approach is expected to significantly improve the precision of ME-BYO indicators for disease risk prediction by accounting for individual variability in health domain interactions and identifying personalized areas for targeted intervention. Comprehensive validation studies with defined primary endpoints (individualized risk prediction accuracy and clinical outcome improvements) and secondary endpoints (intervention targeting effectiveness and healthcare resource optimization) will be essential to establish the clinical efficacy of DEA-enhanced risk stratification before its broader implementation.

We must uphold the fundamental principle that the ME-BYO index should be grounded in substantial scientific evidence while remaining practical and effective in daily preventive medicine practice and daily life, with the advantage of indicators based on easily measurable parameters for individuals to assess themselves. Nevertheless, efforts to enhance the ME-BYO index are indispensable. The original ME-BYO index we proposed is a generalized index for evaluating the overall health status of individuals. It does not encompass all health conditions. Therefore, it is essential to develop multiple “ME-BYO indices” tailored to specific purposes and users, such as basic indices provided by local governments, detailed indices offered by academia, and indices as commercial products. To address potential ME-BYO variant proliferation across governmental, academic, and commercial applications, a comprehensive, light-touch governance framework that maintains the four fundamental domains of metabolic function, locomotor function, cognitive function, and mental resilience, utilizing standardized measurement approaches and validated instruments as a shared core domain set, can be beneficial. This framework would implement precise nomenclature, such as ME-BYO v2.1-Clinical or ME-BYO v2.1-Research, to ensure transparency and comparability across implementations through explicit versioning and naming conventions.

Additionally, the governance structure would require open documentation of scoring algorithms, along with mandatory sensitivity analyses for any modifications, to ensure reproducibility through transparent weighting and aggregation methodologies. Finally, it would be worthwhile to consider establishing a standardized mapping table to the WHO Intrinsic Capacity domains that facilitates international comparison and alignment with global healthy aging frameworks, while preserving the unique ME-BYO conceptual approach. This governance framework strikes a balance between innovation and scientific consistency, ensuring that diverse implementations maintain core compatibility while allowing for context-specific adaptations in clinical, research, and population health applications.

We recognize that digital equity represents a critical implementation challenge, particularly affecting older adults, lower socioeconomic populations, and culturally diverse communities, who may face barriers in smartphone adoption, digital literacy, or the interpretation of culturally appropriate health concepts. We are currently conducting validation studies across diverse demographic groups to ensure the broad applicability of the ME-BYO index on digital devices.
